# Hope and trust in times of Zika: the views of caregivers and healthcare workers at the forefront of the epidemic in Brazil

**DOI:** 10.1093/heapol/czaa042

**Published:** 2020-07-18

**Authors:** Clarissa Simas, Loveday Penn-Kekana, Hannah Kuper, Tereza Maciel Lyra, Maria Elisabeth Lopes Moreira, Maria do Socorro Veloso de Albuquerque, Thália Velho Barreto de Araújo, Ana Paula Lopes de Melo, Corina Helena Figueira Mendes, Martha Cristina Nunes Moreira, Marcos Antonio Ferreira do Nascimento, Camila Pimentel, Marcia Pinto, Sandra Valongueiro, Heidi Larson

**Affiliations:** c1 Department of Infectious Disease Epidemiology, London School of Hygiene & Tropical Medicine, Keppel St, London WC1E 7HT, UK; c2 International Centre for Evidence in Disability, Clinical Research Department, London School of Hygiene & Tropical Medicine, Keppel St, London WC1E 7HT, UK; c3 Fundação Oswaldo Cruz, Instituto Aggeu Magalhães/Fiocruz, Avenida Professor Moraes Rêgo, S/N Cidade Universitária. CEP 50740-465, Recife, PE, Brasil; c4 Department of Social Medicine, Faculty of Medicine, Federal University of Pernambuco, Avenida da Engenharia, S/N, Bloco D, 1º Andar, Cidade Universitária. CEP: 50.740-600 Recife, PE, Brazil; c5 Instituto Nacional de Saúde da Mulher, da Criança e do Adolescente Fernandes Figueira/Fiocruz, Avenida Rui Barbosa, 716 - Flamengo, Rio de Janeiro, RJ 20021-140, Brazil; c6 Postgraduate Programme in public Health, Center of Medical Sciences, Federal University of Pernambuco. Avenida Professor Moraes Rêgo, S/N Hospital das Clínicas, Bloco E, 4o.andar. Cidade Unitersitária, CEP 50.670-901, Recife -PE, Brazil; c7 Postgraduate Programme in Public Health, Center of Medical Sciences, Federal University of Pernambuco, Avenida Professor Moraes Rêgo, S/N Hospital das Clínicas, Bloco E – 4º Andar, Cidade Universitária, CEP: 50.670-901, Recife, PE, Brazil; c8 Núcleo de Saúde Coletiva da Universidade Federal de Pernambuco R. Alto do Reservatório - Alto José Leal, Vitória de Santo Antã - PE-Brasil, 55608-250l

**Keywords:** Hope, trust, Zika virus, microcephaly, congenital Zika syndrome, caregivers, healthcare workers

## Abstract

This article investigates how hope and trust played out for two groups at the forefront of the Zika epidemic: caregivers of children with congenital Zika syndrome and healthcare workers. We conducted 76 in-depth interviews with members of both groups to examine hope and trust in clinical settings, as well as trust in public institutions, in the health system and in the government of Brazil. During and after the Zika epidemic, hope and trust were important to manage uncertainty and risk, given the lack of scientific evidence about the neurological consequences of Zika virus infection. The capacity of healthcare workers and caregivers to trust and to co-create hope seems to have allowed relationships to develop that cushioned social impacts, reinforced adherence to therapeutics and enabled information flow. Hope facilitated parents to trust healthcare workers and interventions. Hope and trust appeared to be central in the establishment of support networks for caregivers. At the same time, mistrust in the government and state institutions may have allowed rumours and alternative explanations about Zika to spread. It may also have strengthened activism in mother’s associations, which seemed to have both positive and negative implications for healthcare service delivery. The findings also point to distrust in international health actors and global health agenda, which can impact community engagement in future outbreak responses in Brazil and other countries in Latin America.

**Key Messages**

- The ability to trust and to co-create hope may have improved the acceptance of interventions against congenital Zika syndrome, a condition characterized by an uncertain prognosis.
- Negative communication between healthcare professionals and caregivers may lead to lower uptake of interventions and poorer mental health among caregivers.
- Mistrust in the intentions of global health efforts in the wake of an epidemic can adversely impact the outbreak response.

## Introduction

Zika became an international emergency in 2015 in Brazil and other countries in Latin America, leaving a trail of thousands of children with microcephaly and other manifestations of congenital Zika syndrome (CZS). These children experience a range of health conditions, often resulting in severe physical, sensory and cognitive impairment (Miranda-Filho *et al.*, 2016). They have high healthcare needs from a range of providers, putting considerable time and emotional pressure on caregivers (Moreira *et al.*, 2018). Yet the impact of the Zika epidemic on families has received little attention, even though research from a variety of settings, including Brazil, shows that parents of severely disabled children are likely to experience depression, anxiety, stress (Santos Oliveira *et al.*, 2017), spousal separation (Joesch and Smith, 1997; Hartley *et al.*, 2010; Lederman *et al.*, 2015) and negative economic consequences due to the direct and indirect costs incurred through attending to their child’s caring needs (Diniz *et al.*, 2017).

In this study, we investigated the impact of the Zika epidemic on caregivers and healthcare workers in Brazil, one of the most socially unequal countries in the world (Góes and Karpowicz, 2017). Brazil was hit by the Zika epidemic during a period of social unrest: generalized mistrust in government, rooted in social and racial inequalities and contributed to political and societal tensions (Diniz, 2016; Diniz *et al.*, 2017). In 2013, mass protests erupted as millions rioted against high inflation, high taxes, corruption and poor public healthcare services (Watts, 2016). These tensions were further heightened during the World Cup and Olympics in Brazil, which were widely seen as emblematic of the larger problem of unrestrained spending by self-serving politicians despite low standards of health care, social inequalities and corruption (Curi, 2013; Gondim, 2016).

The Zika virus arrived silently in Brazil in 2013 and circulated unnoticed for months, in multiple locations, before the first official diagnoses (Passos *et al.,* 2017) and subsequent widespread outbreak. The Zika vector, the *Aedes aegypti* mosquito, is a vector of other infectious diseases endemic to Brazil, including dengue and chikungunya. After decades of failed vector control policy in Brazil *(*Barreto *et al.*, 2011), chronic underfunding of the public unified health system (Sistema Único de Saúde, which was created in 1990 to provide universal health coverage in Brazil) (Castro *et al.,* 2019), and gross neglect of social determinants of infections, the government and politicians were blamed by many members of the public for the new Zika epidemic (Nunes and Pimenta, 2016). The Zika epidemic further exacerbated historic social vulnerabilities and exposed the state structures of neglect to underserved minority populations (Farmer, 2004). Zika disproportionately affected the poorest families in the poorer parts of the country, who had limited access to health services and lived in areas that lacked basic sanitation, which facilitated mosquito reproduction (Diniz, 2016; Diniz *et al.*, 2017). At the same time, the Brazilian government was perceived to have responded poorly in regard to sexual and reproductive rights, including access to contraceptive methods and planned parenthood during the epidemic (Baum *et al.*, 2016).

The scientific and medical understanding of the Zika virus and its neurological consequences has improved since the first case was reported in Brazil in 2013. Still, nearly 7 years after the start of the epidemic, much uncertainty remains (Vouga *et al.*, 2018). The impact on life expectancy and the extent of damage and impairment in the children related to CZS is still unclear, as are the appropriateness and effectiveness of different treatments and interventions. In the absence of a stronger body of evidence, caregivers and healthcare professionals have struggled to identify adequate care regimens, as well as reliable information and support. Moreover, the ongoing social, economic and political turmoil in Brazil is a potential obstacle for the appropriate provision of social support and healthcare services to meet the needs of affected children and their families (Massuda *et al.*, 2018).

The dominant views within health policy offer few insights into the nature and value of health system relationships (Gilson, 2003). This article explores how hope and trust directly mediated interactions and cooperation (and potentially health outcomes) between two groups at the forefront of the Zika epidemic: caregivers of children with CZS and healthcare workers attending to them. The research investigated their trust in public institutions, in the Brazilian health system and government as they were relevant for interactions with health care within a wider social context of epidemic.

## Theories of trust and hope in clinical settings

Trust is a feature of human relationships and matters in the context of health. Trust is rooted in expectations about how the other party will behave and this expectation, in turn, determines whether a person is willing to accept the risk and become vulnerable to another person’s guidance. To trust is an active choice, and it assumes that the trusted party has the trusting individual’s best interest at heart (Larson *et al.*, 2018). In this article, our conceptualization of trust has emerged through an analysis of relevant literature and consultation with experts and it focuses on the following dimensions (Table 1): generalized trust (Rothstein and Stolle, 2008); historical influences on trust (Gamble, 1997; Boulware *et al.*, 2003); political trust (Levi and Stoker, 2000); trust in information (Larson *et al.*, 2018); networks of trust (Stolle, 2001); and external levers of trust (Larson *et al.*, 2018).

**Table 1:** Key dimensions of trust

Trust dimension	Concept associated with dimension	Associated impact on healthcare provision
Generalized trust	Trust is the willingness of individuals to trust other members of a society to solve collective problems	Generalized trust has been said to play an important role in information flows from policy makers and health authorities to members of wider community (Gilson, 2003; Rothstein and Stolle, 2008)
Historical influences on trust	Historical influences such as past systematic abuse and neglect of populations by health and government officials	It can lead to subsequent distrust in health system and health professionals (Gamble, 1997; Boulware *et al.*, 2003); religious and ethnic minorities are often cited in healthcare trust literature as holding the lower levels of trust in healthcare systems and professionals (Corbie-Smith et al., 2002; Halbert et al., 2006)
Political trust	An assessment of trustworthiness of government and particular political actors	When government and politicians are perceived as trustworthy, citizens are more likely to be agreeable to policy and comply to demands (Levi and Stoker, 2000)
Trust in information	Belief that the health information received is truthful and trustworthy	Relies on the trust in source of information (Larson *et al.*, 2018)
Networks of trust	Networks of trust are relational and provide opportunities for the exchange of information that can promote outcomes desirable to group members	Networks of trust can be established when there is enough social capital among members of a given group, e.g. healthcare professionals groups, parents associations (Stolle, 2001)
External influences on trust	Non-medical sources trusted for health advice	Can include friends, family members, religious organizations, alternative health networks (Larson *et al.*, 2018)

*Source:* Prepared by the authors; references shown in the text.

The importance of trust in patient–provider relations for healthcare delivery has been recognized in the health literature and can be considered in different ways (Chandra *et al.*, 2018). Authors such as Larson *et al.* (2018), Gilson (2003) and Rothstein and Stolle (2008) define generalized trust as individual willingness to trust other member or society, which includes trust between healthcare workers and patients. Yet, the act of trusting the health care received is sustained by a complex web of trust relationships influenced by factors beyond dynamics between individuals. For example, the trust relationships between the individuals and a system (e.g. a health system or a political system) are equally important for trust-based cooperation. A health system’s past performance has a historical influence on trust building, particularly in cases of past systematic abuse and neglect of populations (Gamble, 1997; Boulware *et al.*, 2003). Political trust, or perceived trustworthiness of a government, is another lever for the acceptance of health measures (Levi and Stoker, 2000), especially in the context of pandemic response where aversive public health measures might be necessary.

Another dimension of trust critical to healthcare delivery is trust in health information. Believing in received information is dependent on trusting the source of that information (Larson *et al.*, 2018). For this reason, exchange of information and cooperation is intensified within networks of trust (Stolle, 2001) formed when there is enough mutual trust within members of a group involved in healthcare delivery (healthcare workers groups, patients associations). Simultaneously, there can be non-medical sources trusted for health advice (e.g. family, religious organizations), which are here conceptualized as external influences on trust and which can have repercussion for health outcomes.

Trust is therefore a feature of human relationships, and its presence or absence can impact the quality of interpersonal communication, mutual cooperation and dialogue. To that end, research has focused on trust in health care. Yet little attention has been given to the role of hope, although it is the capacity to hope that enables one’s ability to trust (McGeer, 2008). Cooper *et al.* (2014) define hope as the assessment that individuals make of their circumstances and what they can expect for their future. Taussig *et al.* (2013) identified the concept of ‘potentiality’ as an important feature of hope: to imagine or talk about potential is to imagine or talk about that which does not (and may never) exist. Potentiality is therefore a quality perceived as available to human nurturing and direction through which people can create something other than the current reality (Taussig *et al.*, 2013). In this way, potentiality can be understood as the partner to hope.

Within this definition, medical practice requires a certain amount of hope that improvement in the condition is possible. Even when faced with negative news, allowing room for hope can be considered as vital: hope arises from the confidence that everything humanly possible will be done for the good of the patient, with the assurance that the healthcare team is committed, and therefore enables potentiality. Hope ultimately becomes the meeting point between what is possible and what is probable (Cooper *et al.*, 2014). Hope in a possible future can mobilize caregivers, despite the risk and uncertainty, but it requires trust in the person imparting knowledge.

Yet cultivating hope can, paradoxically, have an adverse impact on healthcare service delivery (Table 2). While hope offers a possibility for a better life even in difficult circumstances, to hope is to be reminded of what is not (e.g. medicine offering no cure) and what might never be possible (e.g. a life free from disability) (Mattingly, 2010). This paradox can put an emotional burden on healthcare workers: how can you ‘administer’ or ‘dose’ the right amount of hope? How to deal with patients who reject and challenge clinical diagnoses? How to adequately help patients find hope in the absence of medical cures (Del Vecchio-Good et al., 1994; Mattingly, 1994; 1998)? Anthropological and sociological studies have discussed disruption and despair brought by the lack of hope in patients and families affected by chronic conditions (Kleinman, 1989; Becker, 1994; Frank, 1995). The challenge lies in how to cultivate hope in a way that is bearable, despite its elusive promises, and one which can also be supported in clinical settings, where expensive or adequate care may not be available.

**Table 2:** Key dimensions of hope

Concept of hope within health care setting	Paradoxical dimensions of hope	Associated impact on healthcare provision
Medical care requires hope in improvement through recommended therapeutics (Cooper *et al.*, 2014)	Positive: allowing space for improvements in chronic conditions (e.g. disability) (Mattingly, 2010)	Intervention uptake; positive mental health and coping of caregivers; motivation
In face of uncertainty, caregivers must be invested in ‘potential’ of different outcomes (Taussig *et al.*, 2013)	Negative: sustaining hope that is inconsistent with available resources and clinical settings (Mattingly, 2010)	Rejection of clinical diagnosis if they are negative; caregivers putting more trust in professionals who offer hope, even if unrealistic

*Source:* Prepared by the authors, based on Cooper *et al.* (2014), Taussig *et al.* (2013) and Mattingly (2010).

This challenge of balancing hope and trust was relevant within the 2015/16 Zika epidemic in Brazil, as thousands of children were born with microcephaly, yet clinical data on the likely prognosis and progression were lacking. This aim of this article is to investigate how hope and trust played out for two groups at the forefront of the Zika epidemic: caregivers of children with CZS and healthcare workers.

## Methods

This study was conducted in Brazil by researchers based in both Brazil and the UK. Two divergent locations were selected where efforts to tackle the Zika epidemic were ongoing, and the teams had good access to families of children with CZS and healthcare professionals active during epidemic. The first was Recife City and Jaboatão dos Guararapes, in the State of Pernambuco in Northeast Brazil. This region was considered the epicentre of the Zika outbreak in Brazil. The second selected site was Rio de Janeiro City, in the State of Rio de Janeiro, where Zika was far less rampant and reports of CZS far inferior. The research project used qualitative research methods to explore how sentiments of hope and trust mediated cooperation between two of the most heavily affected groups by Zika epidemic: caregivers of children with CZS and healthcare workers. The methods included: ethnographic observation in social grounds associated with care and treatment of compromised children (hospital waiting rooms, observation of appointments with healthcare professionals, private homes of families affected by Zika) and semi-structured, in-depth interviews with caregivers of children with CZS and healthcare workers supporting the long-term care of affected children.

Interviews were conducted in Recife city and metropolitan area and Rio de Janeiro (Table 3). A total of three interviewers were used in Recife and four in Rio de Janeiro. All interviewers were female Brazilian social scientists from the local region, who were either already experienced or had undergone training by senior researchers in the group, which included role-play exercises and practice with the interview guides. Interviews were conducted in Portuguese and using a topic guide, which had been developed by the research team and pilot tested and adapted where necessary (Supplementary file 1). In Recife, interviews were conducted face to face at participants’ homes. In Rio de Janeiro, interviews were conducted at Fernandes Figueira Institute (IFF/Fiocruz). All interviews were digitally recorded, transcribed and translated into English. To ensure confidentiality, all data were anonymized and all identifiers (such as names or locations) were removed.

**Table 3:** Research participants and locations in the States of Pernambuco and Rio de Janeiro, Brazil (2017)

Research subjects	State of Pernambuco	State of Rio de Janeiro
Healthcare professionals (*n* = 21)	2 obstetricians 1 neonatal physician 2 family and community health specialists 1 nurse 1 psychologist 1 physiotherapist 1 occupational therapist 1 state health surveillance manager Total: 10	1 neonatal physician 1 obstetrician 1 neuro-paediatrician 1 ophthalmologist 1 psychologist 2 neonatal obstetric nurses 1 social worker 1 biologist 1 hospital surveillance professional 1 nursing technician Total: 11
Caregivers of children with CZS (*n* = 55)	17 mothers 5 fathers 3 grandmothers 1 grandfather 2 aunts 1 great grandmother Total: 29	15 mothers 3 grandmothers 7 fathers 1 aunt Total: 26

*Source:* Prepared by the authors.

### Sampling

Mothers and other caregivers (e.g. father, grandmother) of children with CZS and healthcare workers were recruited according to different inclusion criteria for each site. In Pernambuco, participants were caregivers involved or who directly participated in an existing case-control study in Pernambuco, which has been published (Araújo *et al.*, 2018), and who agreed to participate in this research after follow-up. In Rio de Janeiro, caregivers were recruited at two referral hospitals. Fifty-five caregivers were included, which were not necessarily paired (e.g. grandmother and mother of same child). Participants were intentionally sampled to identify a range of subjects in terms of severity of syndrome, age (of the child and of caregiver), ethnicity and socio-economic status. All participants lived in urban areas.

Healthcare professionals were recruited at both the hospital and primary healthcare levels and aimed to include a range of specialists (e.g. ophthalmologists, physiotherapists) per setting, as well as a clinical epidemiologist at each site. Participants had to be working in the care of children with CZS and their families. Twenty-one healthcare professionals were included; health agents were excluded. Sample sizes were defined when data reached saturation: new information was no longer attained and further coding was not feasible (Fusch and Ness, 2015).

### Data analyses

In addition to in-depth interviews, qualitative methods included 12 participant observations from March to November 2017. In Recife, participant observation occurred during visits to caregivers’ homes and to the headquarter of a mother’s association group. In Rio, at Fernandes Figueira Institute (IFF/Fiocruz), participant observation was undertaken both in the waiting room and during consultations. Participant observation enabled the analyses of social interactions that participants may experience without explicitly talking about ( Russell, 2006). Through direct exposure to the social settings in which caregivers and healthcare workers are immersed, researchers learned behaviours and routine activities of study participants. This provided important insight to contextualize data analyses.

NVivo 11 software (QSR International, Melbourne, Australia) was used to conduct deductive thematic analyses. A deductive approach was used, based on the literature reviewed. The interviews were coded in different trust categories: generalized trust, historical influences of trust, political/system trust, trust in information, networks of trust and external influences on trust. Findings were organized under the two mains themes of hope and trust to develop a theory of how they enabled clinical interactions between caregivers and healthcare workers. Two investigators discussed coding categories (LP-K and CS), and one (CS) coded the data (with input from LP-K and HL). Interviews were anonymized, and no real names were used in this article. Researchers ensured that all measures were taken to avoid information to be tracked back to interviewees.

### Ethics

Due to the close links between Zika epidemic and sexual and reproductive rights, sensitive topics such as illegal abortion were expected to arise during interviews. For this, oral consent was chosen in place of written consent to ensure that the information would not be tracked back to participants. This was done to protect women and healthcare professionals who could have partaken in such practices as abortion is a punishable crime in Brazil. Likewise, the datasets generated and analysed during the current study are not publicly available; however, anonymized data can be provided upon reasonable request. Prior to the beginning of interviews, an informed consent sheet was read, and participants gave oral consent, and the responsible researcher signed to testify that it had been done. This study received approval from Research Ethics Committees of the authors’ institutes after following all recommended ethical protocols.

## Results

The Zika epidemic in Brazil cast uncertainty about the future of those children affected by CZS and their caregivers. Understanding experiences of care and issues of trust and hope in this context provides valuable insights for healthcare delivery to these, and similar, groups. All caregivers described an intense care routine for their children with CZS, usually consisting of at least weekly doctor appointments and physiotherapy at varied locations. Despite personal sacrifices and uncertainty of outcomes, most caregivers remained invested in the potential of a less debilitating future for their children. The absence or presence of trust and hope, in difference stances, had important implications for caregivers’ mental health, adherence to treatment and information flow.

### Hope enabling generalized trust between caregivers and healthcare workers

Caregivers of children with disability are likely to face a range of challenges, which can result in a negative impact on their mental health and well-being (Giallo *et al.*, 2013; Kuper *et al.*, 2018). Parents of children with CZS have to deal with the acceptance of the children’ condition and limitations, adjusting to possible relationship conflicts, financial problems and time spent on health services. All of this could increase the likelihood of psychological suffering and lower levels of mental health among the caregivers, notably the mother (de Souza *et al.*, 2018). When trust and hope were present in the relationship between caregivers and healthcare professionals, the negative emotional and social impacts of CZS appear to have been mitigated due to a more open exchange of information, as well as sharing of emotions and feelings. A mother interviewed shared her experience, an example of generalized trust: ‘I said to the doctor: “I am feeling desperate, I cannot lose my son; he is the only child I have and I came to ask for your help.” The doctor told me “don’t worry mum, come tomorrow and we will admit him and have all the exams done (…).” I felt very welcomed, they did all the exams and to this day my son is very well treated here’.

Hoping for their child’s rehabilitation, caregivers trusted healthcare workers, even after professionals admitted uncertainty about the outcomes of interventions. The healthcare workers’ honesty with caregivers, while sharing their hope, helped support the ‘leap of faith’ (Brownlie and Howson, 2005) necessary for trust. Caregivers recurrently trusted healthcare workers who said that they believed in a better future for them and their children and so offered hope, even in the absence of certainty. Those encounters had a positive impact on caregivers; they were presented with possibilities and a potentially different future. Potentiality, brought by the idea of a possible future scenario, allowed space for hope to grow. Caregivers who were offered hope may be more committed to continuing investment in their child at home. As one of the mothers’ state: ‘Everything that the physiotherapists do with her at the clinic, I repeat at home. They tell me “do this with her” and I do it at home. I do it so that she can develop properly, so that she does not have delays of many things’.

### Absence of hope and eroding generalized trust between caregivers and healthcare professionals

Positive examples of hope and trust were not always present. While most caregivers appeared to trust healthcare workers, other interviewees reported negative experiences. They shared painful tales of healthcare workers who, instead of hope, offered gloomy futures. One male expert physician advised a mother: ‘You should not be spending time and money coming to the hospital, you should be saving up to buy his coffin’. Another physician said: ‘I do not know why you keep trying, there is no way your son will escape lying in a bed vegetating for the rest of his life’. The potential consequences of negative communication are profound. An absence of potentiality may have dampened hope and, soon after, trust. In the period of heightened uncertainty in regard to neurodevelopment of these children, a biomedical determinism that gives caregivers a sense of hopelessness appears to have negative emotional and motivational impacts, with consequent lower uptake of interventions and poorer mental health of the caregiver.

There were also more subtle forms of negative communication between providers and caregivers. The potential of improvement, when taken away, was reported by some caregivers as having negative social and emotional consequences on their lives. One caregiver shared her grief in the face of recent news that her son would need a wheelchair. The physiotherapist had said that she should order a wheelchair as soon as possible as it could take months and her son needed it immediately, which triggered intense grief and sorrow in the mother. The perceived potential of a future, one in which her son would walk, had been interrupted abruptly. She frequently described her journey of caring for her son in terms of mournful moments in which imagined futures were no longer possible. This example touches on the issue of hope and sets up challenges for healthcare workers: Should they feed hope, which could be false, which would then break trust, or communicate uncertainty more directly and risk breaking hope and perhaps reducing the trust relationship between the caregiver and healthcare provider?

As trust is co-created between healthcare workers and caregivers, some healthcare workers talked about their own lack of trust in caregivers of children affected by CZS. For instance, some healthcare workers believed that caregivers of children with CZS duped the system to get more than other caregivers of children with severe disability not caused by Zika. Some healthcare workers believe that caregivers took advantage of the media attention and international donors for their own benefit, gathering presents and support that other disabled children do not receive.

### Trust in information in times of scarce evidence

An important issue is that some healthcare professionals struggled to find a realistic approach for giving appropriate information while accepting their own uncertainty about outcomes. One of the mothers interviewed describe the high level of uncertainty: ‘one of the doctors said my daughter would die after birth and another said everything was normal and the microcephaly was nothing to worry about’. It became difficult for caregivers to trust and hope, in the context of inconsistent and negative information. A number of caregivers did not express complete trust in healthcare workers, and some sought additional advice, using private healthcare services whenever possible. They reported sometimes seeking information from healthcare workers who gave them a sense of hope.

The lack of trust in healthcare information among some caregivers and healthcare workers also enabled alternative explanations and rumours about Zika to spread. For instance, a common rumour circulated claiming that microcephaly is caused by an expired batch of measles, mumps and rubella (MMR) vaccines administered by the government. One father said: ‘sometimes I had doubts if the cause was really the mosquito or if it was something else, if it was due to a vaccine …’. This vaccine rumour was also present among healthcare workers, with one of them explaining: ‘No one knew what was happening to the children, and the media was terrorizing everyone saying it was the expired MMR vaccines which caused microcephaly; there was a lot of confusion and we did not know what was happening’.

### External influences on trust and hope in the face of uncertainty around Zika

There were two frequently mentioned sources of information and support that were both trusted and offered hope: God (a key external lever of trust) and other caregivers (who also formed networks of trust, which is discussed in the next section). Religious belief was frequently reported as an important source of hope, arising from a fundamental trust in God. Often, the caregivers referred to the child affected by Zika as their divine mission on Earth. As one mother said: ‘God only sends these special children to special mothers’. Another mother affirmed: ‘my child is teaching me so much, to love more, to be more understanding. God sent him to me so I could change’. They trusted their children to be ‘little angels’ sent by God to teach them about unconditional love. Indeed, one of the biggest Zika-related mother’s associations was named ‘Mothers of Angels’. Trust that there is a divine mission especially assigned to them appeared to offer hope and appease anxiety in the face of adversity brought by disability. At the same time, this trust facilitated emotional attachment and investment in routine care. As one mother said: ‘I know he is not going to be a normal child, right? But I need to have faith and trust that at least he will be something’.

### Networks of trust and hope

Caregivers also frequently found hope and trust among each other, creating networks of trust. All participants interviewed for this research mentioned the use of WhatsApp, and it appears to be the key medium for the formation of networks of trust and information flows, which offer hope for a better future for them and their child. Indeed, mother’s associations were cited as important networks of trust that operate mainly via WhatsApp. All caregivers interviewed participate in WhatsApp groups, such as in UMA (acronym for ‘Mothers of Angels’) and AMAR (Mothers of Rare Families), in Recife, or Lotus, in Rio de Janeiro (Scott *et al.*, 2017). There is intense knowledge exchange through these mediums, and caregivers report it helps to learn how other mothers deal with similar issues.

These networks can interact with healthcare services, both positively and negatively. In general, members of associations report that they trust healthcare workers. However, there is a tacit understanding that current knowledge about Zika is insufficient to fully grasp the extent of damage and identify the best therapeutics. At the same time, there is also mutual understanding that healthcare workers themselves have been learning from the caregivers, who are an important source of information as they characterize the range of symptoms. Consequently, the mothers at the high ranks of leadership in the associations believe that their experiences caring for their children render them as qualified as healthcare workers to decide the best course of treatment. During participant observation, one of the mother leaders was heard describing herself as her son’s speech therapist and physiotherapist and another mother leader affirmed that she advises other mothers on how to adjust the medication against doctors’ directions. Information shared within networks of trust was therefore perceived to be particularly believable and helpful.

### Political mistrust and hopelessness

Yet, there was lack of trust in two sectors critical to the Zika response: the government (political trust) and the scientific community. There is a strong overarching sentiment among both carers and almost all providers that Zika’s negative impacts were a direct consequence of longstanding social injustices. To quote one of the physicians interviewed: ‘the mosquito itself is very democratic, it bites everyone; but I only see poor people with microcephaly babies—I am yet to see a case in the private clinic I work at’. The presumed blame of the government for the Zika epidemic meant that there was a lack of trust in the ability of the government to meet the healthcare and other needs of children and thereby provide a more hopeful future. Absence of trust in government, in systems and in political representatives appeared to have strengthened mothers and patients’ associations, to demand better quality healthcare and secure the rights of the affected children.

Mistrust in international researchers and health actors also surfaced in many interviews and during participant observation. Participants questioned whether they were actually the intended beneficiaries of interventions and research agendas (Ventura, 2016). Both caregivers and healthcare workers signalled mistrust in researchers and expressed an overarching sentiment that things were being done ‘to them’ rather than ‘for them’. One mother said about her experience with a researcher: ‘He was not interested in helping me, most times I feel like I am an animal in a zoo’. To healthcare workers in particular, the lack of international interest in other serious and widespread epidemics in Brazil, such as dengue and chikungunya, reinforced distrust in global health research intentions. Both caregivers and healthcare workers held mistrust that international health actors acknowledged the social injustices, which brought by the Zika epidemic, and there appeared to be little hope among many of the interviewees that global health efforts were in fact aimed at improving the population’s overall condition.

## Discussion

While adequate access to health care is an important issue in Brazil, particularly for families affected by CZS (Albuquerque *et al.*, 2019), this article contributes to this discussion of the importance of a trust-based healthcare system for cooperation between healthcare workers and caregivers. Health systems are inherently relational, and many of its challenges lie in its behavioural and relationship dynamics (Gilson, 2003). Following the Zika outbreak in Brazil, sentiments of trust and hope mediated cooperation between healthcare professionals and caregivers of children with CZS. Networks of trust were highly important, especially among caregivers. There was generally good generalized trust in healthcare providers. However, at times it broke down—particularly when ideas of potentiality and hope were hindered. Political trust was low, resonating with Brazil’s political development prior and at the time of the epidemic. Trust in information varied greatly by source and, at times, was problematic and contributed to the spread of misinformation. The findings in this study suggest that that trust allowed for potentiality and hope.

For healthcare providers and patients, co-creating an alternative, hopeful future can minimize the negativity of a situation and open a window of opportunity to mitigate stress and uncertainty (Tracy and Huffman, 2017). The co-creation of possible futures could allow for the leap of faith necessary for trust to take hold (Brownlie and Howson, 2005). Indeed, the ability to co-create hope among the caregivers and healthcare workers interviewed seemed to be key to whether caregivers trusted particular healthcare workers. The active choice to hope and trust gives agency to caregivers and reduces feelings of powerlessness in the face of Zika. It allows caregivers to make an effort to create, together with healthcare workers, alternative narratives based on optimism and hope in the future (Tengbeh *et al.*, 2018). This is a complex dynamic as in some cases it seemed that women trusted health workers who might be giving them ‘false hope’.

Healthcare workers were not the only source of hope. Trust in religious doctrine (an external lever of trust) and caregiver groups (networks of trust) also played important roles. In particular, WhatsApp was an important means of information diffusion, but only within social networks of trust. Information trust was usually high but depended on source (Larson *et al.*, 2018). It was also occasionally problematic as exemplified by the vaccine rumours discussed in interviews. In other cases, rampant distrust made it difficult to stop the spread of misinformation, including unfounded vaccine rumours (Tengbeh *et al.*, 2018). The spread of this misinformation is troubling. In future epidemic preparedness efforts, including in the likely case of a Zika vaccine being available in the coming years (Barrett, 2018), there is a need to address rumours and build confidence.

The Zika epidemic has left important lessons for Brazilian health policymakers and officials due to low levels of political trust. The deep mistrust in government and the political system in Brazil expressed by both caregivers and healthcare workers is problematic. Trust in the government has long been viewed as a central determinant of individuals’ adherence to health policies recommendation, constraints and rules. While trust can increase a population’s tolerance for invasive or restrictive public health interventions, distrust can provoke antagonism to government policy and even active resistance (Braithwaite *et al.*, 1998; Levi and Stoker, 2000). The risks posed by distrust in government are accentuated in low and middle income countries, such as Brazil, where mechanisms for mass communication are unreliable (e.g. WhatsApp groups were an important as source of health information among the caregivers interviewed in this study), healthcare access is compromised and suspicions are compounded by long legacies of state weakness (Blair, *et al.*, 2017). In such settings where the fabric of trust has been eroded, outbreaks and spill across borders have broader implications for other settings in Latin America.

The mistrust towards international actors, particularly researchers, is also a challenge. There was a common perception among study participants that they were only an object of research while their broader health needs were not being addressed. Indeed, external interventions can reproduce inequality and injustice, particularly among those whose vulnerable lives are at stake (Scheper-Hughes, 1995).

There were important historical influences of trust present during the Zika epidemic in Brazil as populations most affected were those historically more vulnerable to disease. In fact, pandemics have historically disproportionately affected underserved populations, highlighting lines of disadvantage based on race, economic status and gender (DeBruin *et al.*, 2012). Neutral global approaches to resource allocation during pandemic response, preparedness and research, including those for the current Zika outbreak, could perpetuate and possibly increase existing gender, social and health disparities (Harris *et al.*, 2016). Ultimately, response and preparedness can alleviate the burden of Zika only to the extent it works to address the particular risks confronting the disadvantage populations they affect.

Social conditions influence the risk of contracting disease and ability to recover. Social factors can include straightforward conditions such as quality of nutrition and dependence on public transport, but also the presence of dignity-affirming or dignity-denying experiences (DeBruin *et al.*, 2012). In times of global pandemics, when response includes increased availability of funds to research, the scientific community should strive for dignity-affirming experiences for local populations (Harris *et al.*, 2016). Global health research outputs ought to serve not only the scientific endeavour but also the vulnerable populations under study who sustain the hefty burden of disease.

### Limitations

This study has a number of limitations. First, despite the inclusion of a large number of interviews with both patients and providers in this study, the fieldwork was conducted only in two settings in Brazil, which is the fifth most populous country in the world (Harris *et al.*, 2016) and with marked cultural differences between regions. This has implications for the generalizability of study findings—particularly considering differences in the impact of the Zika epidemic across the country. Second, although the study focused on both male and female caregivers, the authors noticed that women were the main providers of care for these children. Further studies should thus aim to bring new perspectives on the gender division of caregiving role and how this can impact or be impacted by hope and trust. Finally, researchers brought their own perspectives to the data collection and analysis, which may have introduced biases, although we tried to overcome this through rigorous training and double coding of transcripts.

## Conclusion

Hope and trust were central to managing uncertainty and risk during and after the Zika epidemic. The ability to trust and to co-create hope permitted relationships to develop between caregivers and healthcare workers that softened social impacts and allowed trusted information sharing and acceptance of interventions. Hope and trust were also important to establishing novel interpersonal dynamics, including the development of caregiver support groups. In contrast, generalized mistrust in government and public institutions allowed rumours and alternative explanations about Zika to spread, fuelling activism in mothers’ associations leading to positive and negative interactions with healthcare services. The intense global attention given to the Zika epidemic raised local suspicions about underlying motivations of international health actors and consequently led to mistrust. These feelings might have been accentuated by the Brazilian ongoing epidemics of dengue and chikungunya, two diseases that were transmitted by the same vector but did not generate global action. Rather than abstract concepts, trust-based and hopeful relationships may directly impact on global responses to future epidemics. If positive relationships are not rebuilt, the distrust in foreign health actors and global health agenda could persist and impact on future outbreak response in the region.

## Supplementary Material

czaa042_Supplementary_File_1
