# Supplementary material for: Hope and trust in times of Zika: the views of caregivers and healthcare workers at the forefront of the epidemic in Brazil
Source: Health Policy Plan. 2020 Jul 18;35(8):953–61. doi: 10.1093/heapol/czaa042 (PMC7553755; doi:10.1093/heapol/czaa042)
Supplement: czaa042_Supplementary_File_1 [file czaa042_supplementary_file_1.docx]

**Interview guide: caregivers of children with Congenital Zika Syndrome (CZS)**

1. Can you share the story of your/your partner’s pregnancy, from the moment you learned about the pregnancy up to the microcephaly/CZS diagnosis?
2. When you learned about the pregnancy (yours or your partner’s), what did you feel? (explore if this was a planned pregnancy).
3. Did you/your partner have pre-natal care during the pregnancy? If yes, in which health care services? How were the appointments? (explore partners/father participation)
4. Were you worried about Zika during your/your partners’ pregnancy? Why? (explore self-care strategies, care for the environment)
5. Who communicated the diagnosis of microcephaly/CZS to you? How did you feel when you received the news?
6. How did your family/partner react to the diagnosis?
7. During your pregnancy, what information about Zika did you received/looked for? (explore different sources of information). Do you think the information was sufficient/trustworthy?
8. What do you think about abortion in cases of pregnant women who had Zika? And abortion in general?
9. Could you share your child’s care routine? How do you feel about this routine? Who else is involved in this? How do they participate?
10. Could you share the support available to you (family, professionals, neighbours, financial support, emotional support) and that you can count on in your current situation with your child?
11. If you have other children, what are the differences in raising them and your child affected by CZS?
12. How is the relationship between siblings/other children and (name of child affected by CZS)?
13. Has your routine changed? In what ways? (explore work, routine, leisure, costs, access to government benefits).
14. What are your unmet needs?
15. Are you part of any WhatsApp group of mother’s associations?
16. Would you like to complement/add anything to this interview?

**Interview Guide: Health Care Workers (HCW)**

1. What do you presently work with? Could you describe your work activities?
2. Do you remember when you first got information about Zika? (Explore timeline; how did the information reach you? By which means?).

1. Are you in direct contact with families and/or babies affected by CZS? If **YES**, could you qualify the type of contact? (if in the health care services).
2. Do you feel Zika has affected your work? In what ways?
3. What is your opinion about the present state of scientific knowledge in regard to Zika, comparing to the first time you learned about the virus? (Explore the interviewees knowledge about the subject, how is this knowledge being built, if he/she participated in any training, and how was the experience. Explore too if they were aware and instructed about sexual transmission).

1. What are your thoughts about the health services currently available to children affected by Zika, their mothers and families affected by Zika? (depending on answer, explore further the specificities of each group).
2. In regard to women who want to get pregnant, do you know how sexual and reproductive health services have been working to prevent Zika? (explore their opinions about what services should exist).
3. What do you think about services available for pregnant women (their partners and families), whether they are worried or not about Zika?
4. Still on sexual and reproductive health, what do you think about the right to abortion? (explore, afterwards, abortion in case of Zika).
5. How do you see that Zika affected:
6. Families and children?
7. Women who got pregnant at the time of sanitary emergency?
8. Women who are pregnant now? And their families (explore partners, grandparents, etc.)?
9. Women who wish to get pregnant?
10. What were the lessons brought by the Zika epidemic?
